# Assessing calibration and bias of a deployed machine learning malnutrition prediction model within a large healthcare system

**DOI:** 10.1038/s41746-024-01141-5

**Published:** 2024-06-06

**Authors:** Lathan Liou, Erick Scott, Prathamesh Parchure, Yuxia Ouyang, Natalia Egorova, Robert Freeman, Ira S. Hofer, Girish N. Nadkarni, Prem Timsina, Arash Kia, Matthew A. Levin

**Affiliations:** 1 Icahn School of Medicine at Mount Sinai, New York, NY USA; 2 cStructure, La Jolla, CA USA; 3 Institute for Healthcare Delivery Science, Icahn School of Medicine at Mount Sinai, New York, NY USA; 4 Department of Population Health Science and Policy, Icahn School of Medicine at Mount Sinai, New York, NY USA; 5 Department of Anesthesiology, Perioperative and Pain Medicine, Icahn School of Medicine at Mount Sinai, New York, NY USA; 6 The Charles Bronfman Institute for Personalized Medicine, Icahn School of Medicine at Mount Sinai, New York, NY USA; 7 The Division of Data Driven and Digital Medicine (D3M), The Department of Medicine, Icahn School of Medicine at Mount Sinai, New York, NY USA

**Keywords:** Epidemiology, Malnutrition

## Abstract

Malnutrition is a frequently underdiagnosed condition leading to increased morbidity, mortality, and healthcare costs. The Mount Sinai Health System (MSHS) deployed a machine learning model (MUST-Plus) to detect malnutrition upon hospital admission. However, in diverse patient groups, a poorly calibrated model may lead to misdiagnosis, exacerbating health care disparities. We explored the model’s calibration across different variables and methods to improve calibration. Data from adult patients admitted to five MSHS hospitals from January 1, 2021 - December 31, 2022, were analyzed. We compared MUST-Plus prediction to the registered dietitian’s formal assessment. Hierarchical calibration was assessed and compared between the recalibration sample (N = 49,562) of patients admitted between January 1, 2021 - December 31, 2022, and the hold-out sample (N = 17,278) of patients admitted between January 1, 2023 - September 30, 2023. Statistical differences in calibration metrics were tested using bootstrapping with replacement. Before recalibration, the overall model calibration intercept was −1.17 (95% CI: −1.20, −1.14), slope was 1.37 (95% CI: 1.34, 1.40), and Brier score was 0.26 (95% CI: 0.25, 0.26). Both weak and moderate measures of calibration were significantly different between White and Black patients and between male and female patients. Logistic recalibration significantly improved calibration of the model across race and gender in the hold-out sample. The original MUST-Plus model showed significant differences in calibration between White vs. Black patients. It also overestimated malnutrition in females compared to males. Logistic recalibration effectively reduced miscalibration across all patient subgroups. Continual monitoring and timely recalibration can improve model accuracy.

## Introduction

Widespread adoption of electronic health records (EHR) has facilitated the implementation of machine-learning based models to enable data-driven outcome prediction^1,2^. Historically, assessment of predictive model performance has focused on discrimination rather than calibration^3–5^. Calibration, the “Achilles’ Heel” of predictive analytics, is defined as the agreement between the predicted probability of an outcome for a particular person and the observed frequency of that outcome among all similar patients^6^. If a model is poorly calibrated, even with high discrimination, it can result in biased individual-predicted probabilities which lead to poorer decision-making by patients and healthcare professionals^7–10^. While an increasing number of studies has developed and validated models based on EHR data for a variety of health outcomes, there is heterogeneity in how calibration is reported, and moreover, only a few have assessed performance of models post-deployment^11,12^. In this study, we systematically assess calibration of an actively deployed malnutrition predictive model (MUST-Plus) that generates dynamic predictions within a three-day window, whether calibration has changed for various patient subgroups since it was first deployed in the Mount Sinai Health System (MSHS), and the performance of recalibrating the model. Identifying cases of malnutrition has important clinical implications because accurate identification and clinical management of malnutrition has been found to reduce risk for hospital-acquired conditions and readmission within 30 days^13–15^. MUST-Plus is derived from EHR data using a Random Forest approach to improve timely identification of high-risk patients to registered dieticians^16^. We hypothesized that the calibration of the MUST-Plus model would not be significantly different between male and female patients nor between White and Black patients.

## Results

The primary training cohort used to recalibrate the model included 49,652 patients (median [IQR] age = 66.0 [26.0]), of which 49.9% self-identified as female, 29.6% self-identified as Black or African American, 54.8% were on Medicare and 27.8% on Medicaid. 11,664 (24%) malnutrition cases were identified. Baseline characteristics are summarized in Table 1 and malnutrition event rates are summarized in Supplementary Table 2. The validation cohort used to test the model included 17,278 patients (median [IQR] age = 66.0 [27.0]), of which 49.8% self-identified as female, 27.1% self-identified as Black or African American, 52.9% were on Medicare, and 28.2% on Medicaid. 4,005 (23%) malnutrition cases were identified.

**Table 1:** Summary of Baseline Characteristics

	No Malnutrition (*N* = 37878)	Malnutrition (*N* = 11774)	Overall (*N* = 49652)	SMD
Age				0.34
Median [IQR]	64.0 (28.0)	70.0 (23.0)	66.0 (26.0)	
Gender				0.16
Female	19606 (51.8%)	5177 (44.0%)	24783 (49.9%)	
Male	18272 (48.2%)	6597 (56.0%)	24869 (50.1%)	
BMI (kg/m2)				0.03
Median [IQR]	27.1 (8.52)	20.7 (4.94)	25.4 (8.80)	
Missing	7250 (19.1%)	2191 (18.6%)	9441 (19.0%)	
Race				0.05
Asian	2465 (6.5%)	835 (7.1%)	3300 (6.6%)	
Black Or African American	11182 (29.5%)	3534 (30.0%)	14716 (29.6%)	
Other	10900 (28.8%)	3146 (26.7%)	14046 (28.3%)	
White	13331 (35.2%)	4259 (36.2%)	17590 (35.4%)	
Ethnicity				0.06
Hispanic	9361 (24.7%)	2612 (22.2%)	11973 (24.1%)	
Not Hispanic/Latino	28517 (75.3%)	9162 (77.8%)	37679 (75.9%)	
Medical/Surgical				0.16
Med	24542 (64.8%)	8475 (72.0%)	33017 (66.5%)	
Surg	13336 (35.2%)	3299 (28.0%)	16635 (33.5%)	
Payor Type				0.25
Commercial	7013 (18.5%)	1370 (11.6%)	8383 (16.9%)	
Medicaid	10887 (28.7%)	2929 (24.9%)	13816 (27.8%)	
Medicare	19766 (52.2%)	7435 (63.1%)	27201 (54.8%)	
Other	25 (0.1%)	6 (0.1%)	31 (0.1%)	
Uninsured	187 (0.5%)	34 (0.3%)	221 (0.4%)	
Year				0.02
2021	20834 (55.0%)	6349 (53.9%)	27183 (54.7%)	
2022	17044 (45.0%)	5425 (46.1%)	22469 (45.3%)	
Facility				0.09
Facility 1	4088 (10.8%)	1075 (9.1%)	5163 (10.4%)	
Facility 2	5698 (15.0%)	1567 (13.3%)	7265 (14.6%)	
Facility 3	15331 (40.5%)	4835 (41.1%)	20166 (40.6%)	
Facility 4	7021 (18.5%)	2227 (18.9%)	9248 (18.6%)	
Facility 5	5714 (15.1%)	2054 (17.4%)	7768 (15.6%)	
Missing	26 (0.1%)	16 (0.1%)	42 (0.1%)	
Type of Hospital				0.08
Community Hospital	9786 (25.8%)	2642 (22.4%)	12428 (25.0%)	
Quaternary Academic Hospital	15331 (40.5%)	4835 (41.1%)	20166 (40.6%)	
Tertiary Acute Care	12735 (33.6%)	4281 (36.4%)	17016 (34.3%)	
Missing	26 (0.1%)	16 (0.1%)	42 (0.1%)	

### Calibration and discrimination

Although the model overall had a c-index of 0.81 (95% CI: 0.80, 0.81), it was miscalibrated according to both weak and moderate calibration metrics, with a Brier score of 0.26 (95% CI: 0.25, 0.26) (Table 2), indicating that the model is relatively inaccurate^17^. It also overfitted the risk estimate distribution, as evidenced by the calibration curve (Supplementary Fig. 1). Logistic recalibration of the model successfully improved calibration, bringing the calibration intercept to −0.07 (95% CI: −0.11, −0.03), calibration slope to 0.88 (95% CI: 0.86, 0.91), and significantly decreasing Brier score (0.21, 95% CI: 0.20, 0.22), Emax (0.03, 95% CI: 0.01, 0.05), and Eavg (0.01, 95% CI: 0.01, 0.02). Recalibrating the model improved specificity (0.74 to 0.93), PPV (0.47 to 0.60), and accuracy (0.74 to 0.80) while decreasing sensitivity (0.75 to 0.35) and NPV (0.91 to 0.83) (Supplementary Tables 2 and 3).

**Table 2:** Overall calibration statistics for MUST-plus model

Calibration Hierarchy	Calibration Metric	No Recalibration (95% CI)	Recalibration In the Large (95% CI)	Logistic Recalibration (95% CI)
Weak	Calibration Intercept	−1.17 (−1.20, −1.14)	0.36 (0.32, 0.40)	−0.07 (−0.11, −0.03)
Weak	Calibration Slope	1.37 (1.34, 1.40)	1.37 (1.34, 1.40)	0.88 (0.86, 0.91)
Moderate	Brier Score	0.26 (0.25, 0.26)	0.21 (0.20, 0.22)	0.21 (0.20, 0.22)
Moderate	Emax	0.26 (0.25, 0.26)	0.22 (0.19, 0.25)	0.03 (0.01, 0.05)
Moderate	Eavg	0.19 (0.19, 0.20)	0.04 (0.03, 0.04)	0.01 (0.01, 0.02)

**Fig. 1:**
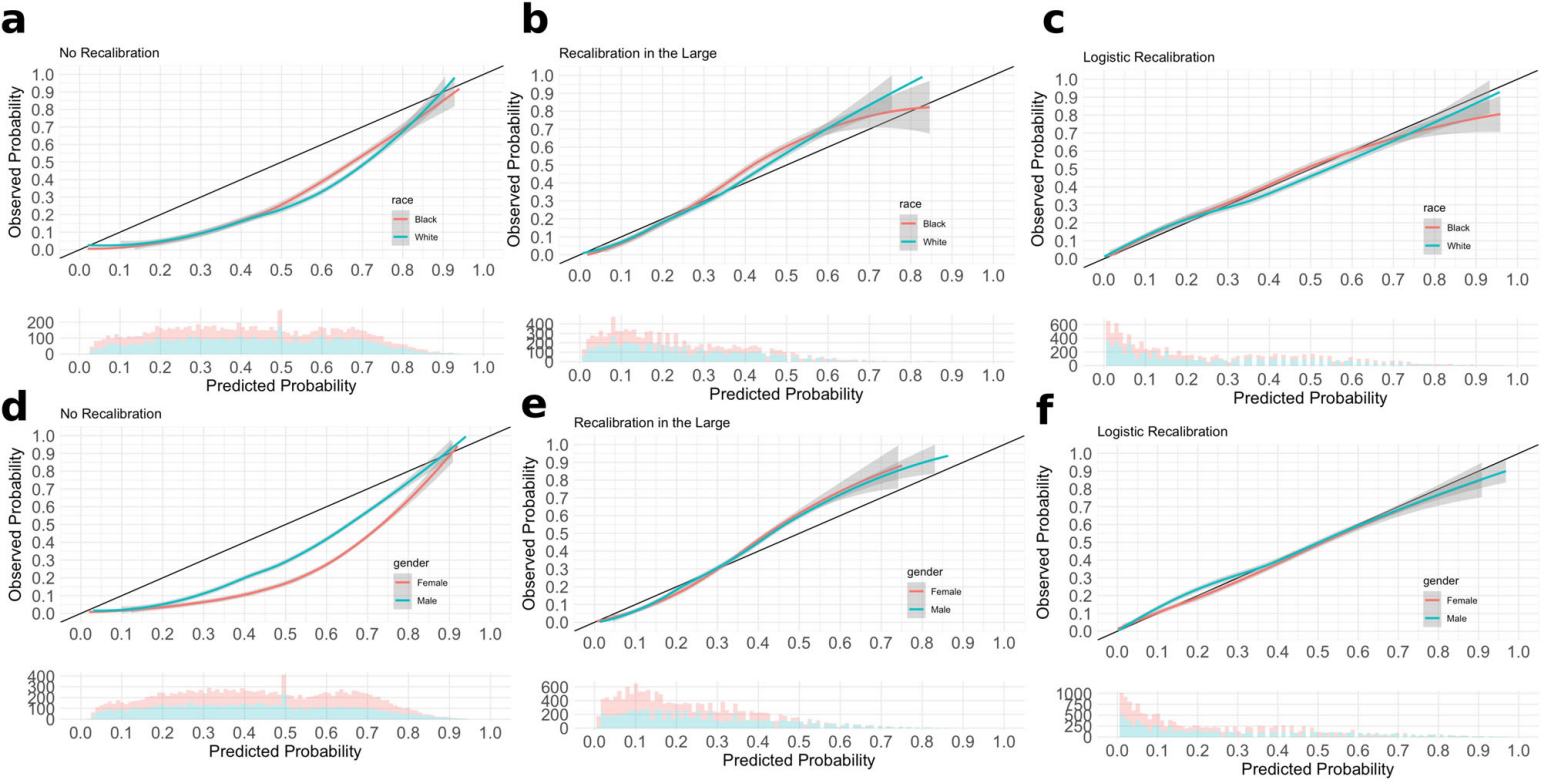
Calibration Curves. Columns from left to right are curves for **a**, No Recalibration **b**, Recalibration-in-the-Large and **c**, Logistic Recalibration for Black vs. White patients **d**, No Recalibration **e**, Recalibration-in-the-Large and **f**, Logistic Recalibration for male vs. female patients.

Weak and moderate calibration metrics between Black and White patients significantly differed prior to recalibration (Table 3, Supplementary Fig. 2A, B), with the model having a more negative calibration intercept for White patients on average compared to Black patients (−1.17 vs. −1.07), and Black patients having a higher calibration slope compared to White patients (1.43 vs. 1.29). Black patients had a higher Brier score of 0.30 (95% CI: 0.29, 0.31) compared to White patients with 0.24 (95% CI: 0.23, 0.24). Logistic recalibration significantly improved calibration for both Black and White patients (Table 4, Fig. 1a–c). For Black patients within the hold-out set, the recalibrated calibration intercept was 0 (95% CI: -0.07, 0.05), calibration slope was 0.91 (95% CI: 0.87, 0.95), and Brier score improved from 0.30 to 0.23 (95% CI: 0.21, 0.25). For White patients within the hold-out set, the recalibrated calibration intercept was -0.15 (95% CI: -0.20, -0.10), calibration slope was 0.82 (95% CI: 0.78, 0.85), and Brier score improved from 0.24 to 0.19 (95% CI: 0.18, 0.21). Post-recalibration, calibration for Black and White patients still differed significantly according to weak calibration metrics, but not so according to moderate calibration metrics and the strong calibration curves (Table 4, Fig. 1). Calibration curves of the recalibrated model showed good concordance between actual and predicted event probabilities, although the predicted risks for Black and White patients differed between the 30th and 60th risk percentiles. Logistic recalibration also improved the specificity, PPV, and accuracy, but decreased the sensitivity and NPV of the model across both White and Black patients (Supplementary Tables 2and 3). Discriminative ability was not significantly different for White and Black patients before and after recalibration. We also found calibration statistics to be relatively similar in Asian patients (Supplementary Table 4).

**Table 3:** Empirical bootstrap differences in calibration intercept and slope before recalibration

Comparison	Calibration Intercept Mean Difference (*P* value)	Calibration Slope Difference (*P* value)	Brier Score Difference (*P* value)	Eavg Difference (*P* value)	Emax Difference (*P* value)
White - Black	**−0.10 (0)**	**−0.14 (2e-04)**	**−0.06 (0)**	**0.03 (0)**	**0.03 (0)**
Female - Male	**−0.61 (0)**	0.01 (0.33)	**−0.07 (0)**	**0.12 (0)**	**0.09 (0)**

Bold denotes statistical significance at Bonferroni-adjusted threshold of 0.001

**Table 4:** Calibration statistics for MUST-plus model by race and gender

	No Recalibration					Recalibration In the Large					Logistic Recalibration				
	Weak Calibration		Moderate Calibration			Weak Calibration		Moderate Calibration			Weak Calibration		Moderate Calibration		
	**Calibration Intercept (95% CI)**	**Calibration Slope (95% CI)**	**Rescaled Brier Score (95% CI)**	**Emax (95% CI)**	**Eavg (95% CI)**	**Calibration Intercept (95% CI)**	**Calibration Slope (95% CI)**	**Rescaled Brier Score (95% CI)**	**Emax (95% CI)**	**Eavg (95% CI)**	**Calibration Intercept (95% CI)**	**Calibration Slope (95% CI)**	**Rescaled Brier Score (95% CI)**	**Emax (95% CI)**	**Eavg (95% CI)**
**Black**	**−1.07 (−1.11, −1.02)**	**1.43 (1.37, 1.49)**	**0.30 (0.29, 0.31)**	**0.23 (0.22, 0.24)**	**0.17 (0.16, 0.18)**	**0.43 (0.36, 0.50)**	**1.43 (1.37, 1.49)**	0.23 (0.21, 0.24)	0.15 (0.10, 0.19)	**0.04 (0.04, 0.05)**	**0 (-0.07, 0.05)**	**0.91 (0.87, 0.95)**	0.23 (0.21, 0.25)	0.08 (0.04, 0.11)	0.01 (0, 0.02)
**White**	**−1.17 (−1.21, −1.12)**	**1.29 (1.23, 1.35)**	**0.24 (0.23, 0.24)**	**0.26 (0.25, 0.27)**	**0.20 (0.19, 0.20)**	**0.25 (0.19, 0.31)**	**1.29 (1.24, 1.34)**	0.20 (0.18, 0.21)	0.20 (0.15, 0.25)	**0.03 (0.02, 0.03)**	**−0.15 (−0.20, −0.10)**	**0.82 (0.78, 0.85)**	0.19 (0.18, 0.21)	0.05 (0.02, 0.09)	0.02 (0.01, 0.03)
**Male**	**−0.88 (−0.92, −0.84)**	1.40 (1.36, 1.45)	**0.29 (0.29, 0.30)**	**0.20 (0.19, 0.21)**	**0.15 (0.14, 0.16)**	0.39 (0.34, 0.44)	1.40 (1.36, 1.45)	0.22 (0.21, 0.24)	0.18 (0.15, 0.21)	0.04 (0.03, 0.05)	**0 (−0.05, 0.03)**	0.88 (0.85, 0.90)	0.23 (0.22, 0.24)	0.04 (0.03, 0.07)	0.02 (0.01, 0.02)
**Female**	**−1.49 (−1.54, −1.45)**	1.42 (1.37, 1.47)	**0.22 (0.21, 0.23)**	**0.32 (0.31, 0.32)**	**0.24 (0.23, 0.24)**	0.40 (0.34, 0.47)	1.42 (1.37, 1.47)	0.20 (0.19, 0.22)	0.24 (0.20, 0.29)	0.04 (0.03, 0.04)	**−0.11 (−0.16, −0.06)**	0.91(0.87, 0.94)	0.21(0.20, 0.22)	0.02 (0.01, 0.05)	0.01 (0, 0.01)

Bold indicates a statistically significant difference in calibration metric between pairs (Black vs. White, Male vs. Female).

Calibration metrics between male and female patients also significantly differed prior to recalibration (Table 3, Supplementary Fig. 2C, D). The model had a more negative calibration intercept for female patients on average compared to male patients (−1.49 vs. −0.88). Logistic recalibration significantly improved calibration for both male and female patients (Table 4, Fig. 1d–f). In male patients within the hold-out set, the recalibrated calibration intercept was 0 (95% CI: −0.05, 0.03), calibration slope was 0.88 (95% CI: 0.85, 0.90), and Brier score improved from 0.29 to 0.23 (95% CI: 0.22, 0.24). In female patients within the hold-out set, the recalibrated calibration intercept was −0.11 (95% CI: −0.16, −0.06), calibration slope was 0.91 (95% CI: 0.87, 0.94), but the Brier score did not significantly improve. After logistic recalibration, only calibration intercepts differed between male and female patients. Calibration curves of the recalibrated model showed good concordance, although the predicted risks for males and females differed between the 10th and 30th risk percentiles. Discrimination metrics for male and female patients were significantly different before recalibration. The model had a higher sensitivity and NPV for females than males, but a lower specificity, PPV, and accuracy (Supplementary Table 2). The recalibrated model had the highest sensitivity (0.95, 95% CI: 0.94, 0.96), NPV (0.84, 95% CI: 0.83, 0.85) and accuracy (0.82, 95% CI: 0.81, 0.83) for female patients, at the cost of substantially decreasing sensitivity (0.27, 95% CI: 0.25, 0.30) (Supplementary Table 3).

We also assessed calibration by payor type and hospital type as sensitivity analyses. In the payor type analysis, we found that malnutrition predicted risk was more miscalibrated in patients with commercial insurance with more extreme calibration intercepts, Emax, and Eavg suggesting overestimation of risk (Supplementary Tables 5 and 6, Supplementary Fig. 3A, B). We did not observe substantial differences in weak or moderate calibration across hospital type (community, tertiary, quaternary) except that tertiary acute care centers had a more extreme calibration intercept, suggesting an overestimation of risk (Supplementary Tables 7 and 8, Supplementary Fig. 3C, D). Across both subgroups, logistic recalibration significantly improved calibration across weak, moderate, and strong hierarchy tiers (Supplementary Table 5, Supplementary Table 7, Supplementary Figs. 4 and 5).

## Discussion

In this study, we evaluated the calibration and discrimination of the MUST-Plus model, a real-time deployed malnutrition prediction model in active use in our health system. The results illustrate that even though discrimination metrics were not significantly different between Black and White patients, model calibration did differ. Furthermore, results demonstrate that the discriminative capacity and calibration of the MUST-Plus model to identify malnutrition significantly differed between male and female subgroups, with exaggerated risks at the tails of the distribution. The negative calibration intercept in the overall model prior to recalibration suggests that malnutrition risk is overestimated, while the positive calibration slope suggests that the risk distribution is underfitted.

MUST-Plus is a model that contains BMI, length of stay, and several laboratory biomarkers such as hemoglobin, serum albumin, serum creatinine, blood urea nitrogen, and serum alanine-aminotransferase as predictors. It has been deployed as an automated EHR-based screening tool, enabling daily assessments for all hospitalized patients. Higher-risk patients are referred to registered dieticians for assessment and treatment as necessary. We hypothesized that the MUST-Plus model would remain stable in its predictive capability throughout its deployment. However, the MUST-Plus model was miscalibrated between 2021 and 2022, which could be due to possible target population shift from time of model development to time of model assessment or the variability of selected predictors from the original model development^3^. This underscores the importance of continual monitoring of predictive model performance such that providers don’t induce patient harm. Matheny et al. found that a deployed acute myocardial infarction predictive model displayed poor calibration, defined using calibration curves, and recommended future recalibration^11^. Sun et al. assessed calibration metrics of a deployed acute kidney injury prediction model and the effects of recalibrating; however, their use case did not use dynamically predicted risk within a time window of event onset, but rather a retrospective labeling^12^.

We found that although the model had similar discriminative capacity between White and Black patients, it was differentially calibrated. This finding requires nuanced interpretation. It is encouraging that the model has had similar discrimination for both White and Black patient populations, meaning that higher risk estimates are calculated for patients with malnutrition than for patients without malnutrition. However, that the model had a higher calibration slope for Black patients indicates that the model was underfitted. Figueroa et al. found that there were no meaningful differences in patient experience between Black and White hospitalized patients, suggestive of broader shifting healthcare quality practices to reduce the health disparities in malnutrition management for hospitalized Black patients^18^. However, other studies have found retrospectively that EHR-based prediction models were miscalibrated and underestimated risk in Black and Hispanic populations^18^. Continuous surveillance such that historically underserved patient populations do not receive biased predicted risk is of utmost importance.

The original model was miscalibrated by overestimating malnutrition risk for female patients compared to male patients. This finding complements those of other single-center cross-sectional studies that find that females are associated with higher risks of malnutrition compared to males, suggesting that detecting malnutrition cases should be preferable^19–21^. It is important to note that Castel et al., Larburu et al., and Arieh et al. defined malnutrition using self-reported outcomes based on either the Mini Nutritional Assessment, Nutritional Risk Index, or Nutritional Risk Screening surveys in cross-sectional studies, so their outcomes may be subject to recall bias. Although logistic recalibration improved calibration of the model for female patients, it decreased sensitivity to 0.27. This suggests that recalibration corrected the overestimation at the expense of producing more false negatives.

Given that the malnutrition rates were similar between the calibration sample and the prospective hold-out sample, decreasing the malnutrition risk prediction thresholds would be expected to improve sensitivity. However, this finding warrants future investigation in systematically modulating prediction risk thresholds and assessing discrimination and calibration. Although overprediction of malnutrition in females can lead to overconfidence in diagnosis, inappropriate treatment, or poor allocation of resources, it is important for providers to remember that women from underserved communities are often doubly vulnerable to malnutrition because of high nutritional requirements during pregnancy or lactation and gender inequalities in poverty^22^. In a sensitivity analysis, we discovered that most calibration metrics differed between patients on commercial insurance compared to those on Medicaid or Medicare. This is likely indicative that payor type serves as a proxy for overall socioeconomic and health status and should not be over-interpreted.

Our results indicate that logistic recalibration was the most effective at improving the model’s calibration, as evidenced by the improvements in various calibration statistics and the graphical representation of calibration curves overall and across different patient subgroups. Mishra et al. notes that in settings where the miscalibration pattern at the risk threshold is similar to the pattern for the bulk of the data, standard logistic recalibration may adequately improve calibration at the risk threshold^23^. While other studies have used recalibration in the large to recalibrate their predictive models, our results show that logistic recalibration generally outperforms the recalibration in the large method^24–26^. While retraining models is also possible and shown to provide improvements in performance^24^, recalibration may sometimes be preferred to maintain predictive context with the original model^23^. Other work has compared standard logistic recalibration to refitting methods^25–27^.

It is essential to acknowledge some limitations of our study. First, our analysis was based on data from a single health system which may limit the generalizability of the findings to other healthcare settings. That said, MSHS is a large health system that encompasses several hospitals which serve uniquely diverse populations in New York City and the broader Northeastern United States. Second, we did not benchmark retraining the MUST-Plus model nor other recalibration methods as that was out-of-scope for our present research study. However, we systematically show that logistic recalibration outperforms recalibration in the large across different subgroups. Third, we did not have more granular data on parity and pregnancy status, which could have enabled more detailed subgroup analyses to better understand the association of females and increased predicted risk.

In conclusion, our study evaluated the calibration and discrimination of the MUST-Plus model, a real-time malnutrition prediction tool based on electronic health record data from the Mount Sinai Health System. We found the model to be differentially miscalibrated between Black and White patients as well as male and female patients; however, logistic recalibration was effective in improving the model’s calibration.

## Methods

### Study population

Institutional Review Board Approval (IRB #18-00573) was obtained prior to beginning this retrospective study. The study cohort consisted of adults (age ≥ 18 years) admitted to the Mount Sinai Health System between January 1, 2021 and September 30, 2023 who had an evaluation performed by a certified registered dietician (RD) as described below. The cohort was split into a training sample of patients admitted between January 1, 2021 and December 31, 2022 and a hold-out validation sample of patients admitted January 1 - September 30, 2023. The RD evaluation was considered the gold standard to which the predicted output was compared. The five individual hospitals assessed were anonymized for analysis.

### MUST-plus model workflow

We sought to assess calibration, in addition to discrimination, of a real-time deployed malnutrition prediction model (MUST-Plus) derived using EHR data from the Mount Sinai Health System. The model has been deployed at five MSHS hospitals since 2020. The details of the model development have been previously described^16^. Briefly, the MUST-Plus model uses a Random Forest approach with 53 parameters to predict the likelihood of moderate or severe malnutrition upon hospital admission. MUST-Plus predictions are generated daily and sent to the EHR (Epic, Epic Systems, Verona, WI). Predicted probability cutoffs used to define a positive prediction vary by site (range 0.44-0.58, Supplementary Table 1). Site-specific thresholds were chosen to achieve the best balance of performance (defined by sensitivity and specificity) at each site.

The MUST-Plus prediction is used by the RD team to prioritize which inpatients to evaluate during their daily rounds. After evaluation, a malnutrition diagnosis (yes/no) is documented by the RD if a minimum of two of the following diagnostic criteria were met: inadequate energy (kilocalorie) intake compared to estimated requirements; significant percentage of unintentional body weight loss within one year; and findings of muscle wasting, subcutaneous fat wasting, or fluid accumulation (edema) on physical examination^16^. If a dietitian sees a patient and confirms the diagnosis of malnutrition, further predictions are permanently suppressed. If no malnutrition is documented, a label of no malnutrition is assigned and predictions are suppressed for three days after which they are again updated and sent to the EHR, and the patient will be seen again based on their priority. For the analysis, we used the maximum predicted probability during the patient admission and then the first RD assessment after that prediction. Only patients with at least one RD evaluation were included in the analysis.

### Calibration analysis

We systematically evaluated calibration of the MUST-Plus model according to the calibration hierarchy proposed by Van Calster et al. and described briefly below^6^. We assessed calibration of the model across various subgroups: race, gender, hospital facility, type of hospital, and payor type. Discrimination was assessed using sensitivity, specificity, positive predictive value, negative predictive value, and Harrell’s concordance index (c-index)^28^. Bootstrapping with replacement (*n* = 100) was performed to generate confidence intervals for discrimination and calibration metrics using the *boot* R package (version 1.3)^29^. Empirical two-sided p-values for differences in calibration intercepts, calibration slopes, Brier scores, Eavg, and Emax were calculated from bootstrapped distributions. The Bonferroni correction was used to adjust for multiple testing.

Weak calibration assesses calibration more broadly by measuring the intercept (optimal = 0) and slope (optimal = 1) of a logistic calibration fit. An intercept greater than 0 indicates an overestimation of risk, and a logistic calibration slope greater than 1 indicates underfitting of the model to the data.

Moderate calibration assesses calibration more carefully by measuring the concordance of predicted risks with observed events using smoothed calibration curves. When moderate calibration is perfectly achieved, the predicted risk loess line falls exactly along the diagonal, meaning that the predicted risk is exactly equivalent to the observed incidence. If the loess line deviates from the diagonal, it indicates an under- or over-prediction of risk. We also calculated other approaches to assessing moderate calibration, such as the rescaled Brier score (range 0-1, lower score better)^7,28^, Eavg (range 0-1, lower score better), and Emax (range 0-1, lower score better). The integrated calibration index (ICI, denoted Eavg) calculates the average absolute difference between the loess predicted risk line and the ideal diagonal, providing a single number to summarize moderate calibration (optimal = 0)^30^. Harrell also proposed using the maximum absolute vertical deviation of the loess predicted risk line and the ideal diagonal (denoted Emax, optimal = 0) as another summary measure for moderate calibration assessment^28^. According to Van Calster et al., moderate calibration is sufficient to provide clinical decision guidance^6^.

Strong calibration assesses moderate calibration across levels of covariates and has been known to be challenging to achieve for many covariates in practice. To visualize some extent of strong calibration, we created calibration curves subset by race, gender, and hospital facility and assessed distribution of predicted risks within each subgroup.

We recalibrated the model on a training set of all patients who were evaluated by the RD team between January 1, 2021 and December 31, 2022, and assessed the performance and calibration of the model on a hold-out set of patients evaluated between January 1, 2023 and September 30, 2023. Recalibration-in-the-large and logistic recalibration approaches were followed as outlined in Vergouwe et al.^27^. Briefly, recalibration-in-the-large was achieved by setting the slope to 1 and estimating the intercept, and logistic recalibration was achieved by allowing the slope and intercept to be freely estimated.

### Supplementary information

Supplementary Information

## Data Availability

Anonymized source data used to generate the results is available upon reasonable request.

## Code availability

Code for analysis can be publicly accessed at https://github.com/latlio/malnutrition_calibration. All analysis was performed using R version 4.3.0^31^ and R Studio version 2023.03.1 + 446^32^. The *rms* package version 6.7^33^ was used to calculate calibration statistics. The number of bootstrap samples (denoted by the *K* variable) is set to 100 by default in the script.
