# Supplementary material for: Assessing calibration and bias of a deployed machine learning malnutrition prediction model within a large healthcare system
Source: NPJ Digit Med. 2024 Jun 6;7:149. doi: 10.1038/s41746-024-01141-5 (PMC11156633; doi:10.1038/s41746-024-01141-5)
Supplement: Supplementary file 1 — Supplementary Information [file 41746_2024_1141_MOESM1_ESM.pdf]

Assessing Calibration and Bias of a Deployed Machine Learning Malnutrition Prediction Model within a Large Healthcare System

Lathan Liou MPhil[1], Erick Scott MD[2], Prathamesh Parchure MS[3], Yuxia Ouyang[4,5], Natalia Egorova[4], Robert Freeman RN[3], Ira S. Hofer MD[5,6,7], Girish N Nadkarni MD[6,7], Prem Timsina PhD[3], Arash Kia MD[3,5], Matthew A Levin MD[3,5,6]

Supplementary Information

Tables

Supplementary Table 1. Site-specific predicted probability thresholds
Supplementary Table 2. Event rates and discrimination metrics before calibration
Supplementary Table 3. Discrimination metrics of logistic recalibration on hold-out set
Supplementary Table 4. Calibration statistics for MUST-Plus model for Asian patients
Supplementary Table 5. Calibration statistics for MUST-Plus model by type of payor
Supplementary Table 6. Pairwise empirical bootstrap differences in calibration intercept and slope before recalibration for type of payor
Supplementary Table 7. Calibration statistics for MUST-Plus model by type of hospital
Supplementary Table 8. Pairwise empirical bootstrap differences in calibration intercept and slope before recalibration by type of hospital

Figures

Supplementary Figure 1. Calibration curves for overall model.
Supplementary Figure 2. Histograms of bootstrapped calibration intercepts and calibration slopes across race and gender
Supplementary Figure 3. Histograms of bootstrapped calibration intercepts and calibration slopes across payor and hospital types
Supplementary Figure 4. Calibration curves for different payor types
Supplementary Figure 5. Calibration curves for different hospital types

Supplementary Table 1. Site-specific predicted probability thresholds

Facility	Cutoff
1	0.46
2	0.49
3	0.58
4	0.45
5	0.44

Supplementary Table 2. Event rates and discrimination metrics before recalibration

Grouping	Proportion of Malnutrition (# Cases)*	Sensitivity (95% CI)	Specificity (95% CI)	PPV (95% CI)	NPV (95% CI)	Accuracy (95% CI)
**Overall**	0.24 (11664)	0.75 (0.74, 0.76)	0.74 (0.73, 0.75)	0.47 (0.46, 0.48)	0.91 (0.90, 0.92)	0.74 (0.73, 0.75)
**White (N=17590)**	0.24 (4259)	0.75 (0.73, 0.76)	0.73 (0.72, 0.74)	0.47 (0.45, 0.48)	0.90 (0.89, 0.91)	0.73 (0.72, 0.74)
**Black (N=14716)**	0.24 (3534)	0.76 (0.74, 0.78)	0.75 (0.74, 0.76)	0.49 (0.47, 0.51)	0.91 (0.90, 0.92)	0.75 (0.74, 0.76)
**Male (N=24869)**	0.26 (6597)	0.73 (0.71, 0.74)	0.77 (0.76, 0.78)	0.53 (0.52, 0.55)	0.89 (0.88, 0.90)	0.76 (0.75, 0.77)
**Female (N=24783)**	0.21 (5177)	0.78 (0.76, 0.79)	0.71 (0.70, 0.72)	0.41 (0.40, 0.43)	0.92 (0.91, 0.93)	0.72 (0.71, 0.73)

PPV = positive predictive value; NPV = negative predictive value

***Proportion of malnutrition is calculated with subgroup as denominator, e.g. proportion of white malnutrition cases = 0.24 = 4225/17487**

Supplementary Table 3. Event Rates and Discrimination Metrics After Logistic Recalibration

Grouping	Sensitivity (95% CI)	Specificity (95% CI)	PPV (95% CI)	NPV (95% CI)	Accuracy (95% CI)
**Overall**	0.35 (0.33, 0.37)	0.93 (0.92, 0.94)	0.60 (0.58, 0.62)	0.83 (0.81, 0.84)	0.80 (0.78, 0.81)
**White (N=17590)**	0.34 (0.32, 0.37)	0.93 (0.91, 0.94)	0.58 (0.55, 0.62)	0.82 (0.81, 0.84)	0.79 (0.78, 0.80)
**Black (N=14716)**	0.39 (0.36, 0.42)	0.93 (0.91, 0.94)	0.62 (0.58, 0.66)	0.83 (0.82, 0.85)	0.80 (0.79, 0.82)
**Male (N=24869)**	0.43 (0.40, 0.45)	0.91 (0.90, 0.92)	0.64 (0.62, 0.67)	0.82 (0.80, 0.83)	0.78 (0.77, 0.80)
**Female (N=24783)**	0.27 (0.25, 0.30)	0.95 (0.94, 0.96)	0.59 (0.55, 0.63)	0.84 (0.83, 0.85)	0.82 (0.81, 0.83)

PPV = positive predictive value; NPV = negative predictive value

Supplementary Table 4. Calibration statistics for MUST-Plus model for Asian patients

Calibration Hierarchy	Calibration Metric	No Recalibration (95% CI)	Recalibration In the Large (95% CI)	Logistic Recalibration (95% CI)
Weak	Calibration Intercept	-1.66 (-1.79, -1.53)	0.44 (0.29, 0.59)	-0.07 (-0.18, 0.03)
Weak	Calibration Slope	1.59 (1.45, 1.75)	1.59 (1.45, 1.75)	0.98 (0.89, 1.08)
Moderate	Brier Score	0.12 (0.10, 0.15)	0.24 (0.20, 0.27)	0.25 (0.22, 0.29)
Moderate	Emax	0.35 (0.33, 0.37)	0.40 (0.32, 0.47)	0.12 (0.05, 0.19)
Moderate	Eavg	0.26 (0.24, 0.28)	0.05 (0.04, 0.07)	0.02 (0.01, 0.03)

Supplementary Table 5. Calibration statistics for MUST-Plus model by type of payor

		**No Recalibration**			**Logistic Recalibration**		
**Calibration Hierarchy**	**Calibration Metric**	**Medicare (95% CI)**	**Medicaid (95% CI)**	**Commercial (95% CI)**	**Medicare (95% CI)**	**Medicaid (95% CI)**	**Commercial (95% CI)**
Weak	Calibration Intercept	-1.05 (-1.08, -1.01)	-1.15 (-1.20, -1.10)	-1.71 (-1.79, -1.63)	-0.07 (-0.11, -0.03)	0.03 (0.02, 0.06)	-0.23 (-0.33, -0.13)
Weak	Calibration Slope	1.36 (1.32, 1.40)	1.36 (1.30, 1.43)	1.40 (1.30, 1.50)	0.88 (0.85, 0.91)	0.89 (0.84, 0.93)	0.90 (0.84, 0.97)
Moderate	Brier Score	0.24 (0.23, 0.25)	0.28 (0.27, 0.29)	0.26 (0.25, 0.27)	0.22 (0.21, 0.23)	0.17 (0.15, 0.19)	0.24 (0.21, 0.27)
Moderate	Emax	0.23 (0.22, 0.24)	0.25 (0.23, 0.26)	0.36 (0.34, 0.37)	0.06 (0.03, 0.09)	0.03 (0.02, 0.06)	0.10 (0.05, 0.17)
Moderate	Eavg	0.18 (0.17, 0.18)	0.19 (0.18, 0.20)	0.25 (0.24, 0.26)	0.01 (0.01, 0.02)	0.02 (0.01, 0.03)	0.02 (0.01, 0.03)

Supplementary Table 6. Pairwise empirical bootstrap differences in calibration intercept and slope before recalibration for type of payor

	Weak Calibration		Moderate Calibration		
**Comparison**	**Calibration Intercept Mean Difference (P-Value)**	**Calibration Slope Difference (P-value)**	**Brier Score Difference (P-value)**	**E_{avg} Difference (P-value)**	**E_{max} Difference (P-value)**
Medicaid - Commercial	**0.56 (0)***	-0.03 (0.28)	0 (0.13)	**-0.06 (0)***	**-0.11 (0)***
Medicare - Commercial	**0.66 (0)***	-0.04 (0.25)	**0.01 (0)***	**-0.08 (0)***	**-0.12 (0)***
Medicare - Medicaid	**0.11 (0)***	0 (0.47)	**0.01 (0)***	0 (0.003)	-0.01 (0.01)

* and bold denotes statistical significance at Bonferroni-adjusted threshold of 0.001

Supplementary Table 7. Calibration statistics for MUST-Plus model by type of hospital

		No Recalibration			Logistic Recalibration		
**Calibration Hierarchy**	**Calibration Metric**	**Community Hospital (95% CI)**	**Tertiary Acute Care (95% CI)**	**Quaternary Academic (95% CI)**	**Community Hospital (95% CI)**	**Tertiary Acute Care (95% CI)**	**Quaternary Academic (95% CI)**
Weak	Calibration Intercept	-1.06 (-1.11, -1.01)	-1.39 (-1.44, -1.34)	-1.19 (-1.23, -1.14)	-0.03 (-0.09, 0.04)	-0.21 (-0.26, -0.15)	-0.04 (-0.09, 0)
Weak	Calibration Slope	1.37 (1.30, 1.44)	1.44 (1.37, 1.50)	1.40 (1.35, 1.45)	0.84 (0.79, 0.88)	0.98 (0.93, 1.02)	0.89 (0.85, 0.92)
Moderate	Brier Score	0.28 (0.27, 0.29)	0.25 (0.24, 0.26)	0.25 (0.24, 0.26)	0.15 (0.12, 0.17)	0.27 (0.26, 0.29)	0.23 (0.21, 0.24)
Moderate	Emax	0.22 (0.21, 0.24)	0.29 (0.28, 0.30)	0.26 (0.25, 0.27)	0.10 (0.04, 0.15)	0.05 (0.04, 0.06)	0.02 (0.01, 0.04)
Moderate	Eavg	0.18 (0.17, 0.19)	0.22 (0.21, 0.23)	0.19 (0.18, 0.20)	0.02 (0.01, 0.03)	0.02 (0.01, 0.03)	0.01 (0.01, 0.02)

Supplementary Table 8. Pairwise empirical bootstrap differences in calibration intercept and slope before recalibration by type of hospital

	Weak Calibration		Moderate Calibration		
**Comparison**	**Calibration Intercept Mean Difference (P-Value)**	**Calibration Slope Difference (P-value)**	**Brier Score Difference (P-value)**	**E_{avg} Difference (P-value)**	**E_{max} Difference (P-value)**
Tertiary - Community	**-0.34 (0)***	0.06 (0.09)	0.01 (0.13)	**0.04 (0)***	**0.07 (0)***
Quaternary - Community	**-0.13 (0)***	0.02 (0.28)	**0.01 (0)***	**0.01 (4e-4)***	**0.04 (0)***
Quaternary - Tertiary	**0.20 (0)***	-0.04 (0.17)	0 (0.26)	**-0.03 (0)***	**-0.03 (0)***

* and bold denotes statistical significance at Bonferroni adjusted threshold of 0.001

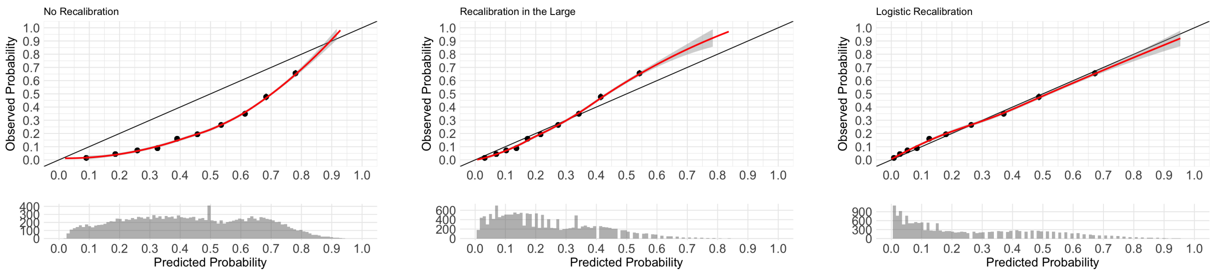

Supplementary Figure 1. Calibration curves for overall model. Columns from left to right are curves for No Recalibration, Recalibration-in-the-Large, and Logistic Recalibration.

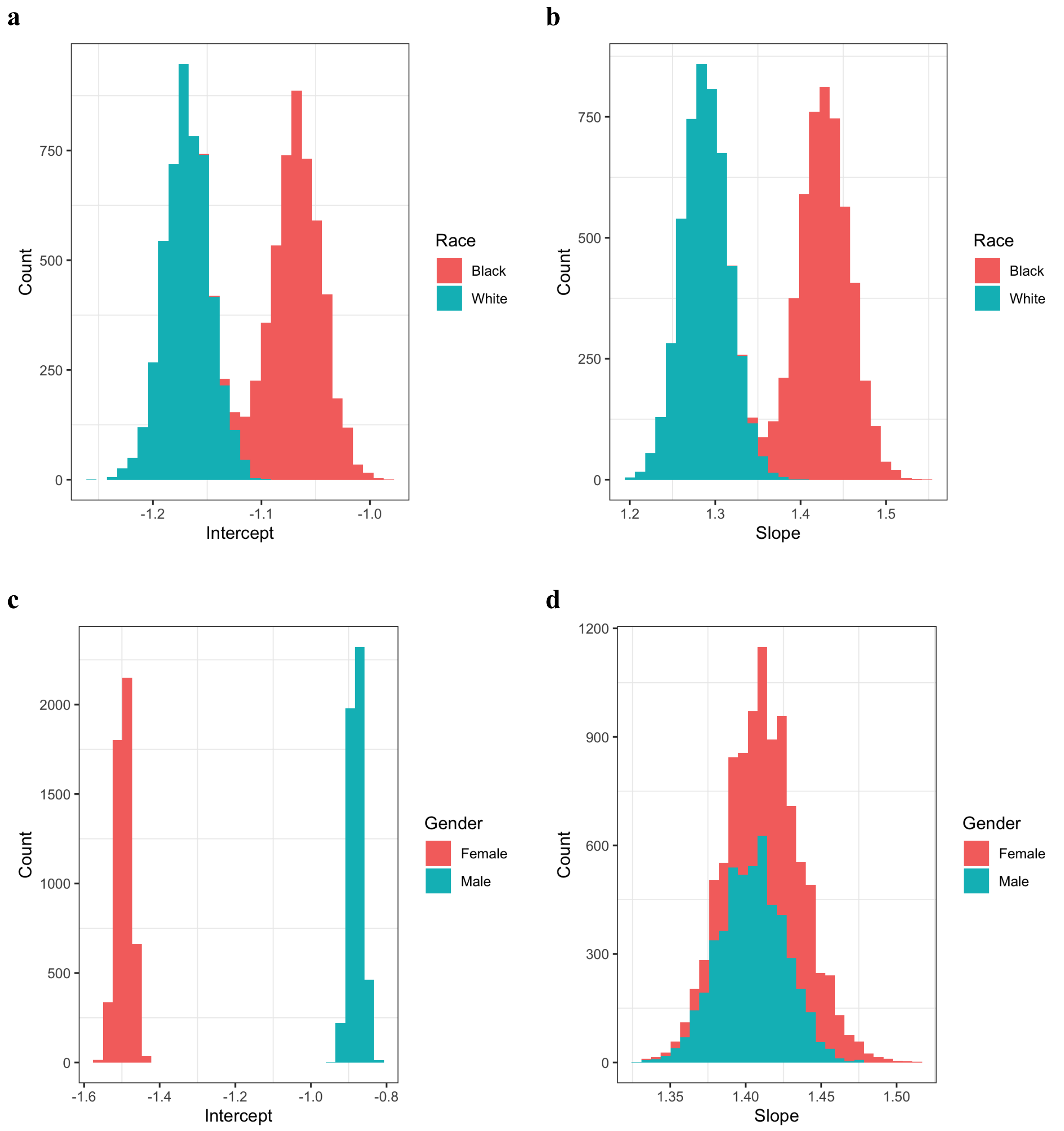

Supplementary Figure 2. Histograms of bootstrapped calibration intercepts (*left column)* and calibration slopes (*right column)* for Black vs. White patients (a-b) and male vs. female patients (c-d)

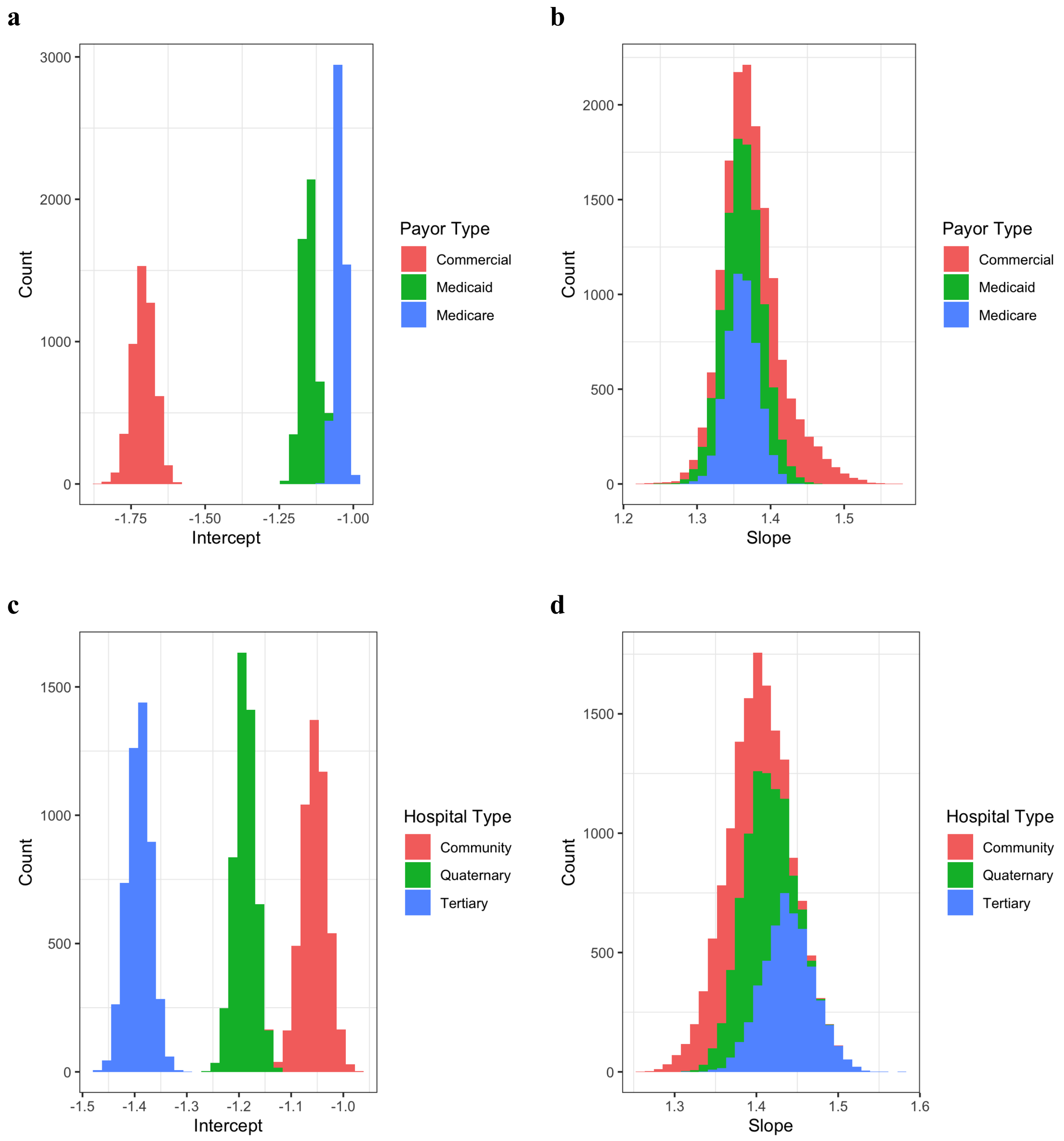

Supplementary Figure 3. Histograms of bootstrapped calibration intercepts (*left column)* and calibration slopes (*right column)* for payor types (a-b) and hospital types (c-d).

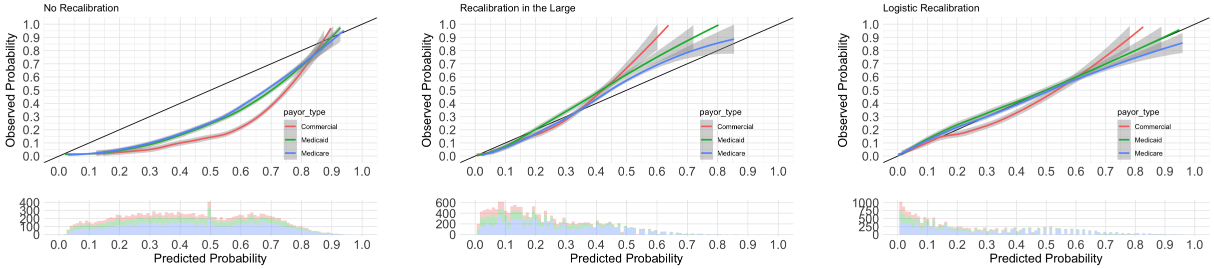

Supplementary Figure 4. Calibration curves for different payor types. Columns from left to right are curves for No Recalibration, Recalibration-in-the-Large, and Logistic Recalibration.

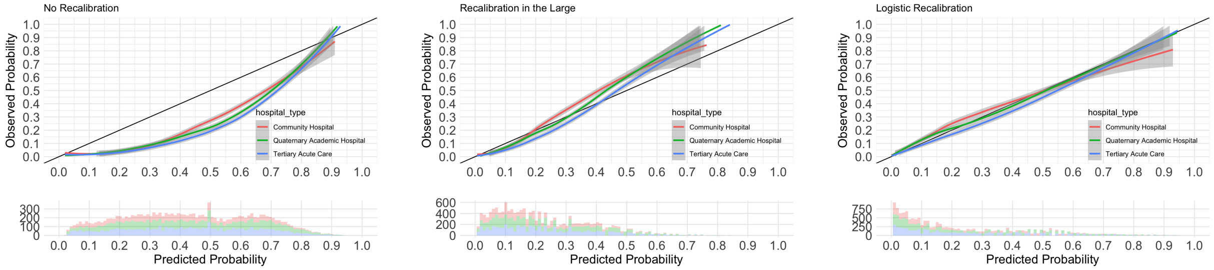

Supplementary Figure 5. Calibration curves for different hospital types. Columns from left to right are curves for No Recalibration, Recalibration-in-the-Large, and Logistic Recalibration.
